# Compartment-specific inflammation in tuberculous lymphadenitis: a case-control study

**DOI:** 10.1007/s00011-026-02351-1

**Published:** 2026-09-05

**Authors:** Caian L. Vinhaes, Samyra Almeida da Silveira, Beatriz Barreto-Duarte, Bruno B. Andrade, Flávia Marinho Sant’Anna, Carolina Arana Stanis Schmaltz, Felipe M. Ridolfi, Valeria C. Rolla, Adriano Gomes-Silva

**Affiliations:** 1 Clinical Research Laboratory on Mycobacteria (LAPCLIN-TB), National Institute of Infectious Diseases Evandro Chagas, Oswaldo Cruz Foundation, Rio de Janeiro, Brazil; 2 Multinational Organization Network Sponsoring Translational and Epidemiological Research (MONSTER) Initiative, Salvador, Brazil; 3 Instituto de Pesquisa Clínica e Translacional, Medicina Zarns, Clariens Educação, Salvador, Brazil; 4 Laboratory of Bacteriology and Bioassays for Tuberculosis and other Mycobacterioses, National Institute of Infectious Diseases Evandro Chagas, Oswaldo Cruz Foundation, Rio de Janeiro, Brazil; 5 Laboratório de Pesquisa Clínica e Translacional, Instituto Gonçalo Moniz, Fundação Oswaldo Cruz, Salvador, Brazil; 6 Division of Infectious Diseases, Department of Medicine, Johns Hopkins University, Baltimore, USA; 7 Department of International Health, Bloomberg School of Public Health, Johns Hopkins University, Baltimore, USA; 8 Interdisciplinary Laboratory of Medical Research, Oswaldo Cruz Institute, Oswaldo Cruz Foundation, Rio de Janeiro, Brazil

**Keywords:** Extrapulmonary tuberculosis, Immune response, Lymph node, Peripheral blood

## Abstract

Extrapulmonary tuberculosis (EPTB) represents a substantial proportion of tuberculosis cases, particularly among people living with HIV. Tuberculous lymphadenitis is one of the most common forms of EPTB and represents a condition in which the lymph node functions both as an immune-organizing site and a focus of *Mycobacterium tuberculosis* (Mtb) infection. Although peripheral blood biomarkers are frequently used to investigate immune responses in tuberculosis, they may not accurately reflect immunological processes occurring at the site of disease. In this study, we aimed to characterize and compare systemic and local inflammatory responses in individuals with suspected lymph node tuberculosis. We conducted a case–control study including 24 individuals undergoing lymph node biopsy for pathological lymphadenopathy at the National Institute of Infectious Diseases Evandro Chagas (Fiocruz), Brazil. Eleven participants were diagnosed with tuberculous lymphadenitis (45.8%) and thirteen served as non-TB controls (54.2%). Paired plasma and lymph node interstitial fluid samples were collected, and concentrations of 29 cytokines, chemokines, and growth factors were quantified using multiplex Luminex assays. Statistical analyses included non-parametric comparisons, hierarchical clustering, Spearman correlation–based network analysis, and canonical correlation analysis to evaluate inflammatory profiles and immune network organization. Plasma inflammatory profiles did not distinguish EPTB cases from controls. In contrast, lymph node samples from EPTB participants exhibited significantly higher concentrations of IL-8, IL-3, and IL-5 and reduced IL-10 levels. Network analyses of correlations between protein concentrations revealed dense and highly interconnected inflammatory networks in plasma, centered on IL-6, IFN-α2, and IL-12p70, whereas lymph node networks were sparser and dominated by TNF-α and IL-1 family cytokines. Cross-compartment analysis identified associations between systemic mediators, particularly TNF-β and IL-7, and pro-inflammatory signals in lymph nodes. Additionally, HbA1c levels were correlated with concentrations of multiple inflammatory mediators, particularly within lymph nodes. Together, these findings indicate that tuberculous lymphadenitis is characterized by marked compartmentalization of immune responses, emphasizing the importance of tissue-level immune profiling for understanding EPTB pathogenesis.

**Supplementary Information:**

The online version contains supplementary material available at https://doi.org/10.1007/s00011-026-02351-1.

## Introduction

Tuberculosis (TB) remains one of the leading public health challenges worldwide, ranking among the infectious diseases with the highest global morbidity and mortality [1]. Although pulmonary disease is the most recognized form, extrapulmonary tuberculosis (EPTB) accounts for a substantial proportion of cases, particularly in people living with HIV (PLHIV) [1, 2]. Among EPTB forms, tuberculous lymphadenitis is one of the most frequent presentations and represents a unique clinical condition in which the lymph node becomes both a site of adaptive immune orchestration and an active focus of *Mycobacterium tuberculosis* (Mtb) infection [2, 3].

The immune response to Mtb infection is complex and involves coordinated interactions between innate and adaptive immunity [4, 5, 6], highlighting the central role of tumor necrosis factor (TNF)-α, interleukin (IL)-12, IL-1β, and interferon (IFN)-γ in macrophage activation and granuloma formation [6, 7]. Effective containment of Mtb depends on a balanced interaction between pro-inflammatory effector mechanisms and regulatory pathways that limit immunopathology [4, 5, 7]. Disruption of this balance can lead either to bacterial persistence or tissue damage [5].

Peripheral blood is frequently used as an accessible source for evaluating immunological biomarkers in patients with TB. Studies based on blood samples have identified cytokine profiles, lymphocyte phenotypes, and transcriptional signatures associated with active disease [8, 9]. Blood transcriptional signatures, particularly those related to interferon pathways and systemic inflammation, have been proposed as diagnostic and treatment-monitoring tools [8, 9]. However, evidence suggests that peripheral blood may not fully reflect immunological events occurring at infection sites such as the lung or lymph node, reinforcing the concept of compartmentalized immune responses [4, 10].

Recent high-dimensional profiling studies have reinforced the concept of immunological heterogeneity in EPTB, identifying distinct inflammatory trajectories and immune phenotypes associated with disease severity [11]. Nevertheless, most investigations rely on peripheral samples or exclude PLHIV, limiting the understanding of how local tissue responses are organized in real-world clinical populations. Additionally, previous studies have provided evidence of compartmentalized immune responses within lymph nodes in EPTB [12, 13, 14].

Given that the lymph node is both a central immune organ and a site of Mtb persistence, comparative analysis of systemic and local inflammatory responses may clarify mechanisms underlying disease manifestation and progression. We recently found no robust distinction between pulmonary TB and EPTB based on plasma levels of cytokines, chemokines, and soluble lipidic mediators [15]. Here we investigated paired plasma and lymph node interstitial fluid samples from individuals undergoing lymph node biopsy to characterize (i) compartment-specific inflammatory activation, (ii) immune network organization within each compartment, and (iii) interactions between systemic and tissue-level responses. By integrating quantitative biomarker profiling and correlation-based network analysis, we sought to determine whether tuberculous lymphadenitis is defined by a distinct inflammatory architecture at the site of disease and how this local response relates to systemic immune activation.

## Materials and methods

### Study design and setting

This case-control study enrolled participants with suspected lymphadenopathy from 2020 to 2022, at the Clinical Research Laboratory on Mycobacteria (LAPCLIN-TB) of the National Institute of Infectious Diseases Evandro Chagas (INI), Oswaldo Cruz Foundation (FIOCRUZ), Rio de Janeiro, Brazil. LAPCLIN-TB has maintained an active TB research cohort since 2000, which operates under a well-established protocol that includes baseline interviews and the collection of demographic, socioeconomic, clinical (including comorbidities), and laboratory data.

### Eligibility criteria

Adult participants aged ≥ 18 years were eligible for inclusion if they presented with pathological lymphadenopathy, had clinical suspicion of tuberculous lymphadenitis, and underwent lymph node biopsy as part of the diagnostic investigation. Participants who had started anti-tuberculosis treatment for the current disease episode more than seven days before lymph node biopsy and blood collection were not included, in order to minimize potential treatment-related effects on inflammatory biomarker measurements.

A previous history of tuberculosis was not considered an exclusion criterion. Major comorbidities other than HIV infection were also not considered exclusion criteria. However, pregnant or breastfeeding women and participants receiving systemic corticosteroids or TNF-α inhibitors were not included.

### Participants and samples

After written informed consent was obtained, all participants with suspected tuberculous lymphadenitis had their clinical, demographic, and epidemiological data recorded in a structured database. A lymph node fragment obtained by surgical excision was investigated for EPTB. The specimen was subdivided into four portions: one for microbiological analysis (culture for *Mtb* and Xpert MTB/RIF Ultra assay), one for histopathological examination, one for mycological investigation, and a fourth portion for immunological analyses.

The diagnosis of tuberculous lymphadenitis was established based on microbiological confirmation (positive Xpert MTB/RIF Ultra assay and/or Mtb culture), histopathological findings compatible with TB, or a combination of compatible clinical presentation and histopathological findings suggestive of TB. Participants were followed prospectively in the outpatient clinic for 12 months, including monthly visits during the first six months of anti-tuberculosis treatment. Clinical response was assessed through improvement of constitutional and local symptoms, reduction in lymph node size, and overall clinical improvement during follow-up. In cases without microbiological confirmation, the diagnosis was further supported by favorable clinical response to anti-tuberculosis treatment and cure at the end of follow-up.

The lymph-node fragment allocated for immunological analyses was placed in sterile phosphate-buffered saline (PBS) immediately after surgical excision and transported to the laboratory under sterile conditions. The tissue fragment was then transferred to a sterile Petri dish containing a pre-weighed stainless-steel mesh (0.3-mm, 50-mash metallic tissue screen). The mesh containing the tissue fragment was weighed again, and the difference between both measurements was used to determine the exact tissue mass. Subsequently, 1 mL of sterile PBS was added to the tissue, which was mechanically dissociated using a sterile pestle until complete disruption of the fragment and release of soluble components into the fluid phase. The resulting suspension was transferred to a cryovial and centrifuged at 500 × g for 10 min at 4 °C. The supernatant obtained after centrifugation was operationally defined as lymph node interstitial fluid. It was aliquoted to avoid repeated freeze-thaw cycles and stored at − 80 °C until biomarker quantification.

In parallel, a peripheral blood sample was collected in an EDTA-containing tube for plasma separation. After processing, plasma samples were stored at − 80 °C for later quantification of soluble mediators. Additional analyses were performed using peripheral blood samples as part of routine clinical laboratory testing (HbA1c, CD4 count and viral load, for PLHIV).

### Biomarker measurement

Cytokine, chemokine and growth factor concentrations were measured in paired plasma and lymph-node interstitial fluid samples using the MILLIPLEX MAP Human Cytokine/Chemokine - Premixed 29-Plex assay (Merck, Hesse, Germany) according to the manufacturer’s instructions. This comprehensive panel allowed for the simultaneous quantification of the following analytes: EGF, EOTAXIN, G-CSF, GM-CSF, IFN-α2, IFN-γ, IL-10, IL-12(p40), IL-12(p70), IL-13, IL-15, IL-17, IL-1RA, IL-1α, IL-1β, IL-2, IL-3, IL-4, IL-5, IL-6, IL-7, IL-8, IP-10, CCL-2, CCL-3, CCL-4, TNF-α, TNF-β, and VEGF. Data acquisition was performed using. the Luminex Intelliflex xMAP technology. Plasma biomarker concentrations were reported as pg/mL. For lymph-node interstitial fluid measured concentrations were normalized by the corresponding tissue mass and expressed as pg/mg of lymph-node tissue.

### Data analysis

The median values with interquartile ranges (IQR) were used as measures of central tendency and dispersion. Cytokine levels were compared between the study groups using the Mann-Whitney U test to identify the biomarkers that were statistically significant between groups. Categorical variables were compared using Fisher’s exact test. Hierarchical cluster analyses (Ward’s method), with z-score normalized data were employed to depict the evaluated biomarkers in the study groups. Dendrograms represent Euclidean distance. *P*-values were adjusted for multiple measurements using Holm-Bonferroni’s method. All univariate comparisons with *P*-values < 0.05 after adjustment were considered statistically significant. Only adjusted* P*-values were considered.

The Spearman rank correlation was used to identify associations between different cytokines and the lymphocyte CD4^+^ T-cell counts of HbA1c percentage. In network analysis based on Spearman correlations, nodes represent each given marker, and lines represent statistically significant correlations (correlation coefficient [rho] >+/-0.5 and *P* < 0.05), focusing on moderate-to-strong associations.

A discriminant model using sparse canonical correlation analysis (CCA) was employed to assess whether a combination of biomarker expression patterns could discriminate between the groups. Our experience with biomarker studies has shown that not only the concentration of a given marker, but also its correlation profile with other markers, is important for evaluating its involvement in a biological phenomenon. In this analysis, differences between study groups were inferred from how each biomarker correlated with the others, rather than from concentration values, alone.

The statistical analyses were performed using GraphPad Prism 11.0.0 (84) (GraphPad Software, La Jolla, CA, USA), JMP 14.0 (SAS, Cary, NC, USA), and R statistical software.

### Ethics statement

The research project was approved by the Ethics and Research Committee of the National Institute of Infectious Diseases Evandro Chagas (CAAE: 07035519.0.0000.5262). Written informed consent was obtained from all participants or their legally responsible guardians, and all clinical investigations were conducted according to the principles expressed in the Declaration of Helsinki.

## Results

### Characteristics of the study population

Twenty-four patients presenting with pathological lymphadenopathy were included in the study. All patients underwent surgical lymph node biopsy and etiological investigation. Microbiological, mycological, and histopathological examinations were performed on all tissue samples. TB investigation was negative in 13 participants and positive in 11 (Table 1).

**Table 1:** Characteristics of the study population

Characteristics	Control (*n* = 13)	EPTB (*n* = 11)	*p*-value
Age – years	40 (28.5–56.5)	45 (30.0–57.0)	0.9
Female sex, n (%)	2 (15.4)	7 (63.6)	0.1
PLHIV, n (%)	5 (38.4)	6 (54.5)	0.6
CD4 count (cells/µL) median	503 (180–845)	288 (100–511)	0.2
HIV viral load, copies/mL median	48 (48–77)	48 (48-15135)	0.9
*TB diagnosis*			
Xpert MTB/RIF Ultra	n.a	6	n.a
LJ Mtb Culture	n.a	4	n.a
Histopathological	n.a	7	n.a

Categorical data were compared between the clinical groups using the Fisher’s exact test; n.a. - not applicable; LJ - Löwenstein-Jensen; PLHIV, people living with HIV

Among the 11 participants diagnosed with tuberculous lymphadenitis, six had positive Xpert MTB/RIF Ultra results, four had positive *M. tuberculosis* cultures, and seven exhibited histopathological findings compatible with TB (Table 1). Two participants had concordant positive results across all three diagnostic methods. One participant was diagnosed based exclusively on positive *M. tuberculosis* culture, whereas three were diagnosed based on histopathological findings alone, including one case in which acid-fast bacilli were identified on tissue examination. Finally, one participant had negative microbiological and histopathological investigations; however, the clinical presentation was highly suggestive of TB, and the patient demonstrated marked clinical improvement after initiation of anti-tuberculosis therapy, ultimately achieving cure at the end of treatment. There were no significant differences between the TB and control groups regarding age, sex, HIV prevalence, or CD4⁺ T-cell counts and HIV viral load among PLHIV (Table 1). Levels of HbA1c were measured only in the EPTB group, with a median [IQR] of 5.6 [5.2–5.8] (data not shown).

Among the 13 non-TB participants included in the control group, nine received a diagnosis based on histopathological examination. Lymphoproliferative disorders represented the most frequent diagnostic category, accounting for 6 of 13 controls (46.1%), including one case of Castleman disease. The remaining participants were diagnosed with reactive or other non-infectious lymphadenopathy (5/13, 38.5%) or acute bacterial infection (2/13, 15.4%). Detailed histopathological descriptions and differential diagnoses are presented in Table 2.

**Table 2:** Presentation of non-TB participants (*n* = 13)

Diagnosis	Histopathologic description	Age	Sex	HIV status
HHV-8 positive Castleman disease	Lymph node with a reactive pattern, showing regressed lymphoid follicles, plasmacytosis, and interfollicular vascular proliferation.	27	Male	Positive
Proteus mirabilis infection	Not performed. Gram-negative bacillus growth in culture.	40	Female	Negative
B-cell lymphoma	High-grade B-cell lymphoma.	61	Male	Positive
Underlying immunological disorder requiring further investigation	Scant lymph node fragment with a reactive pattern. No granulomas identified. No evidence of neoplasia.	20	Male	Not informed
Lymphoma	Differential diagnosis was between classical Hodgkin lymphoma and EBV-positive diffuse large B-cell lymphoma. The available immunohistochemical markers did not allow definitive classification.	70	Male	Negative
Not defined	Lymph node with a reactive pattern. No granulomas identified. No evidence of neoplasia. Special stains for mycobacteria and fungi were negative in the sample.	34	Male	Negative
Not defined	Fragments of lymph node with a reactive pattern, showing follicular hyperplasia and mild eosinophilia. No evidence of neoplasia. No granulomas identified. Special stains for fungi and mycobacteria were negative.	54	Male	Positive
Not defined	Showing fibrosis and collections of histiocytes, occasionally forming small granulomas.	45	Male	Negative
Enterobacter spp. infection	Acute and chronic inflammatory process with foreign body–type giant cells involving the subcutaneous tissue. Special stains for mycobacteria and fungi were negative in the sample.	30	Male	Negative
Small lymphocytic lymphoma / chronic lymphocytic leukemia (SLL/CLL)	Small lymphocytic lymphoma / chronic lymphocytic leukemia (SLL/CLL).	59	Male	Negative
Not defined	Architectural effacement and focal necrosis, showing atypical lymphoproliferation. No granulomas identified.	53	Female	Negative
High-grade lymphoma	High-grade lymphoma.	38	Male	Positive
Not defined	Cervical cyst containing liquid/purulent material, of long-standing duration, with no evidence of an infectious etiology.	24	Male	Negative

Data are presented as median (interquartile range). The Mann-Whitney test was used to compare the levels of each plasma marker between the study groups. P-values in bold font are statistically significant after Holm-Bonferroni adjustment

### *Mycobacterium tuberculosis* infection triggers altered inflammatory profiles in lymph nodes but not in plasma

**Fig. 1:**
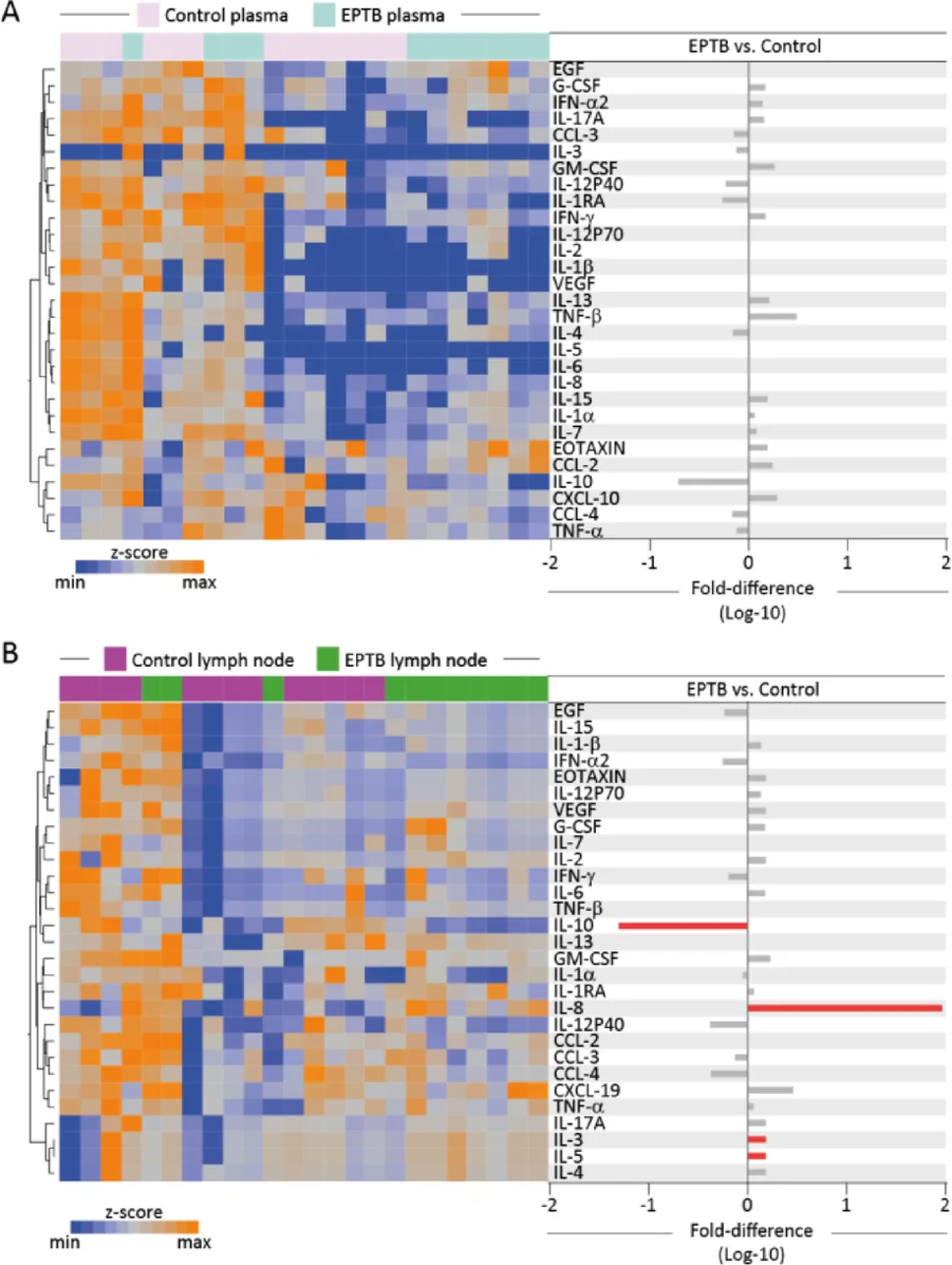
An attenuated inflammatory activation characterizes lymph node tuberculosis. Concentrations of several markers of inflammation were assessed in plasma and lymph node samples from 11 participants with EPTB and 13 controls with other lymph node diseases. Data were log10-transformed. **A** *Left panel*: Hierarchical cluster analysis using z-scored values of each parameter (Ward’s method) in plasma was used to depict the overall biomarker expression profile in the study population. *Right panel*: Fold differences in plasma biomarker concentrations were calculated; no statistically significant differences were identified. **B** *Left panel*: Hierarchical cluster analysis using z-scored values of each parameter (Ward’s method) in lymph node samples was used to depict the overall biomarker expression profile in the study population. *Right panel*: Fold differences in lymph node biomarker concentrations were calculated, and statistically significant differences are highlighted in colored bars. Log10 fold-changes were calculated as EPTB relative to controls; positive values indicate higher concentrations in EPTB, and negative values indicate higher concentrations in controls

To investigate tissue-specific inflammatory activation in EPTB, we compared the concentrations of 29 soluble inflammatory mediators measured in paired plasma and lymph node interstitial fluid samples from the same participants (Fig. 1; Tables 3 and 4). Using z-score–normalized plasma values, hierarchical clustering did not discriminate EPTB patients from controls (Fig. 1A; Table 3).

**Table 3:** Soluble factor concentrations in plasma

Soluble factors (pg/mL)	Control (*n* = 13)	EPTB (*n* = 11)	*p*-value
EGF	72.5 (15.2–132)	112.5 (87.5-130.5)	0.22
EOTAXIN	146.8 (88.5-306.3)	238.3 (198.3-468.8)	0.25
G-CSF	18.5 (7.7–41.2)	27 (13–80)	0.30
GM-CSF	7.5 (3.5–23)	14.5 (5.5–27.5)	0.59
IFN-α2	10 (6-23.5)	15 (9-56.5)	0.17
IFN-g	9 (3-44.7)	14 (7-32.5)	0.50
IL-10	100 (10.3-171.5)	18.5 (10.5–27.5)	0.18
IL-12p40	16 (11-29.7)	9 (7–40)	0.59
IL-12p70	14 (3.7–20.7)	14 (3.7–41.5)	0.49
IL-13	4.2 (3.2–68.2)	7.25 (4.2–24.2)	0.63
IL-15	6.5 (1.7–23.5)	10.5 (4-17.5)	0.57
IL-17 A	12.5 (3.7–23.5)	8.5 (3.7–36.5)	0.75
IL-1RA	25 (9–42)	13 (4–40)	0.55
IL-1α	14.5 (3.2-168.3)	17.5 (8.5–36.5)	0.55
IL-1β	11.1 (11.1–32.2)	11.1 (11.1–36.7)	0.78
IL-2	9 (2.5–16.5)	8.5 (2.5–32.5)	0.57
IL-3	18.2 (18.2–18.2)	18.2 (18.2–18.2)	0.34
IL-4	6 (1.25–150.8)	4 (1.2–42)	0.95
IL-5	10.5 (10.5–41.7)	10.5 (10.5–38)	0.90
IL-6	20.7 (9.8-194.3)	21.7 (9.8–56.2)	1.00
IL-7	5.5 (2.5–22.2)	7 (3.5–19.0)	0.63
IL-8	42 (24.2–362)	44.5 (35-123.5)	0.44
CXCL-10	579 (334.3–2197)	1172 (718.5–1540)	0.49
CCL-2	697 (431.5–1516)	1272 (696–2087)	0.09
CCL-3	12.2 (1.7–89.5)	8.7 (2.2–31.2)	0.63
CCL-4	165 (70-875.5)	108 (73.5–287)	0.41
TNF-α	73 (33-186.8)	52.5 (38.5–80)	0.78
TNF-β	5 (2.7–198)	16 (3.5–31.5)	0.63
VEGF	7.2 (7.2–29.5)	7.2 (7.2–15)	0.62

Data are presented as median (interquartile range). The Mann-Whitney test was used to compare the levels of each plasma marker between the study groups. P-values in bold font are statistically significant after Holm-Bonferroni adjustment.

**Table 4:** Soluble factor concentrations in lymph node interstitial fluid

Soluble factors (pg/mg)	Control (*n* = 13)	EPTB (*n* = 11)	*p*-value
EGF	159,091 (53,079–287,736)	87,500 (79,545 − 175,000)	0.83
EOTAXIN	35,156 (26,042–98,438)	56,250 (51,136 − 80,357)	0.15
G-CSF	45,455 (28,595 − 101,887)	50,000 (45,455 − 325,000)	0.09
GM-CSF	31,250 (23,148–222,349)	50,000 (45,455–575,000)	0.10
IFN-α2	93,750 (37,847 − 268,809)	50,000 (45,455 − 71,429)	0.41
IFN-g	90,909 (30,331–537,247)	50,000 (45,455-1,150,000)	0.94
IL-10	6,734,375 (438,971 − 14,656,945)	262,500 (238,636–958,333)	**0.02**
IL-12p40	62,500 (15,625 − 533,019)	25,000 (22,727 − 208,333)	0.83
IL-12p70	132,076 (107,230–416,193)	187,500 (170,455 − 267,857)	0.25
IL-13	306,035 (28,472–796,875)	212,500 (147,727 − 712,500)	0.72
IL-15	79,545 (50,041–272,727)	87,500 (79,545 − 152,778)	0.38
IL-17 A	117,188 (67,050–178,977)	187,500 (144,231 − 187,500)	0.07
IL-1RA	14,838,889 (5,051,137 − 49,693,966)	28,611,111 (9,900,000–49,423,077)	0.55
IL-1α	204,546 (40,972 − 478,774)	175,000 (34,091–525,000)	0.67
IL-1β	389,151 (318,117-1,200,627)	556,250 (505,682-2,212,500)	0.14
IL-2	78,125 (57,870 − 125,000)	125,000 (96,154 − 125,000)	0.18
IL-3	570,313 (326,309–871,023)	912,500 (701,923 − 912,500)	**0.04**
IL-4	39,063 (28,935 − 59,659)	62,500 (48,077 − 62,500)	0.05
IL-5	328,125 (187,739 − 501,136)	525,000 (403,846 − 525,000)	**0.04**
IL-6	448,864 (299,518-7,004,363)	705,357 (448,864-1,387,500)	0.53
IL-7	34,091 (21,446 − 133,992)	37,500 (28,846 − 100,000)	0.65
IL-8	562,500 (332,031 − 5,765,625)	54,300,000 (2,615,385 − 154,000,000)	**0.007**
CXCL-10	50,875,000 (13,075,000-228,000,000)	153,000,000 (16,634,615 − 498,000,000)	0.30
CCL-2	66,711,111 (44,408,423 − 149,500,000)	66,350,000 (26,045,455 − 119,000,000)	0.86
CCL-3	6,218,750 (1,552,517 − 34,174,465)	15,762,500 (240,385 − 65,312,500)	0.63
CCL-4	178,000,000 (58,889,063–410,000,000)	77,350,000 (13,607,143–146,000,000)	0.11
TNF-α	4,708,333 (920,496 − 12,593,040)	4,375,000 (2,763,889 − 13,425,000)	0.95
TNF-β	22,727 (15,165 − 100,216)	25,000 (19,231 − 25,000)	0.94
VEGF	226,563 (207,312–362,500)	362,500 (329,546 − 362,500)	0.11

Data are presented as median (interquartile range). The Mann-Whitney test was used to compare lymph node interstitial fluid concentrations of each marker between the study groups.* P*-values in bold font are statistically significant after Holm-Bonferroni adjustment.

In contrast, analysis of lymph node–derived biomarkers revealed a tendency toward segregation between EPTB participants and controls (Fig. 1B; Table 4). This pattern was characterized by higher concentrations of IL-8, IL-3, and IL-5 in EPTB lymph nodes, accompanied by lower levels of IL-10. In a sensitivity analysis restricted to participants with lymphoproliferative disorders in the control group, these findings remained unchanged (Supplementary Fig. 1). Together, these results suggest that inflammatory responses in EPTB are more readily detected at the tissue level than in systemic circulation, supporting the presence of compartmentalized immune activation within affected lymph nodes.

### *Mycobacterium tuberculosis* leads to consistent alterations in correlations between concentrations of inflammatory biomarkers in lymph nodes

**Fig. 2:**
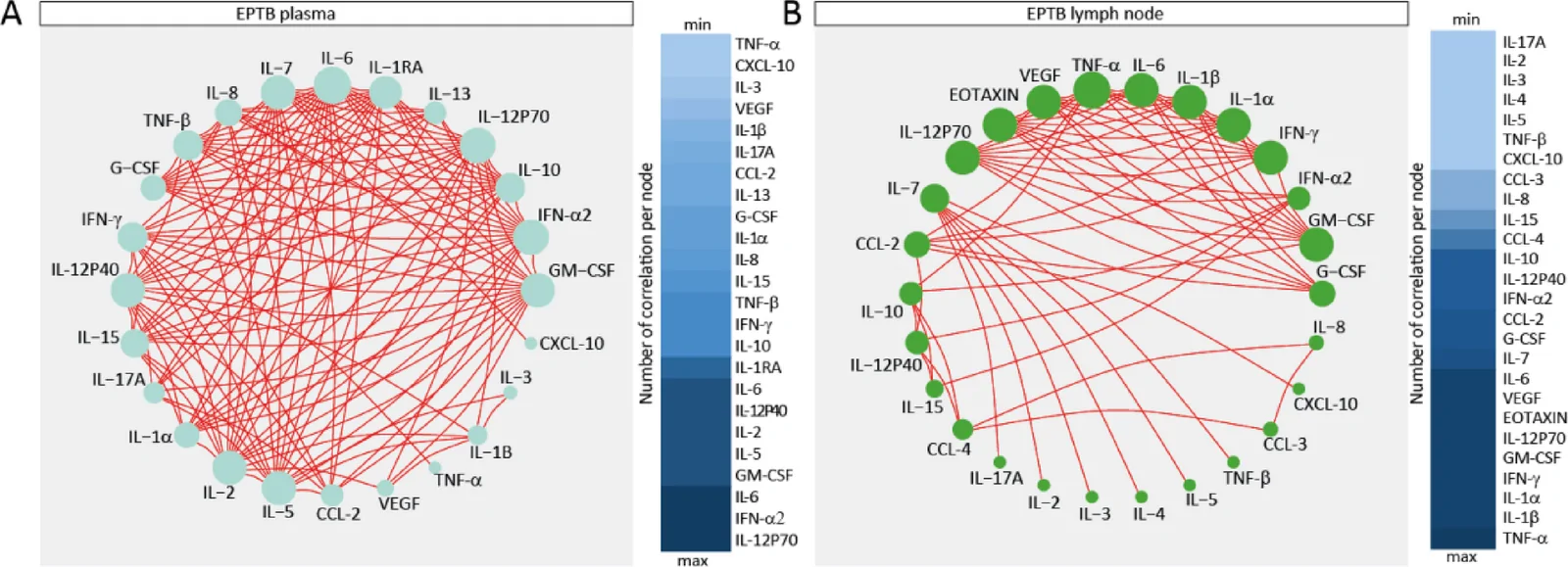
Compartment-specific inflammatory network organization in tuberculosis. **A** Peripheral blood (plasma) and **B** lymph node interstitial fluid. Network analysis of the biomarker correlation matrices was performed with bootstrap (100×). Significant correlations (*P* < 0.05) and Spearman rank correlations (rho) > 0.5 are shown. Each node represents a different parameter. The size of each circle (node) is proportional to the number of significant correlations involving that node. Connecting lines represent Spearman rank correlation coefficient (rho) values. Red lines indicate positive correlation. Color maps on the right of each network denote the hierarchical number of significant correlations per parameter (node) in each clinical group

To further characterize compartment-specific immune activation in EPTB, we performed Spearman correlation analyses to explore the relationships between concentrations of inflammatory mediators, an approach that has been shown to enable the assessment of immune network structure and inflammatory dynamics [16, 17, 18, 19] (Fig. 2).

In plasma, EPTB participants exhibited a dense and highly interconnected correlation network (Fig. 2A). Central nodes included IL-6, IFN-α2, and IL-12p70, indicating a prominent pro-inflammatory axis. Subsequently, IL-7, IL-12p40, IL-2, IL-5, and GM-CSF were the higher connected nodes.

In contrast, analysis of lymph node samples revealed a markedly sparser interaction network (Fig. 2B), characterized by a reduced number of statistically significant correlations between biomarkers. TNF-α emerged as the most connected node, followed by IL-1β, IL-1α, IFN-γ, GM-CSF, IL-12p70, EOTAXIN, VEGF, and IL-6. Importantly, chemokines involved in lymphocyte trafficking and granuloma organization (CCL-2, CCL-3, and CCL-4), as well as cytokines associated with immune regulation and response balance (IL-2 and IL-10), displayed a low number of interactions.

Together, these findings suggest a less interconnected inflammatory network within the EPTB tissue compartment, characterized by a reduced number of statistically significant correlations between inflammatory mediators. This pattern is consistent with altered immune organization at the site of disease, although the functional consequences of this network architecture cannot be determined from the present data.

### Systemic versus local inflammatory responses against *Mycobacterium tuberculosis*

**Fig. 3:**
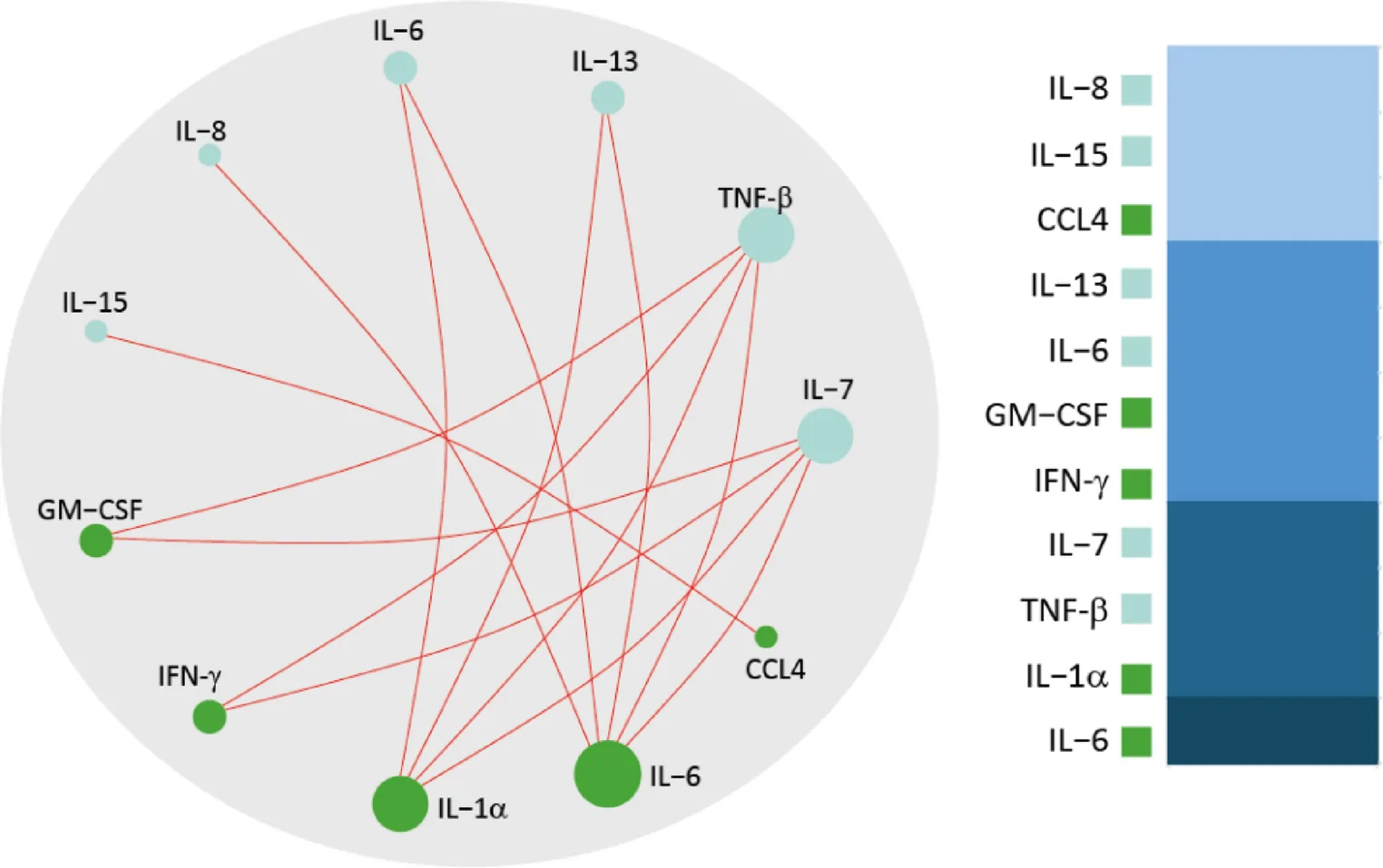
Interactions between plasma and lymph node concentrations of soluble factors’ in EPTB participants. Network analysis of the biomarker correlation matrices was performed with bootstrap (100×). Significant correlations (*P* < 0.05) and Spearman rank correlations (rho) > 0.5 are shown. Each node represents a biomarker, and node size is proportional to the number of significant correlations involving that biomarker. Connecting lines represent significant Spearman correlation coefficients. Red lines denote positive correlations. Blue nodes represent plasma biomarkers, whereas green nodes represent lymph node interstitial fluid biomarkers. The color map displayed to the right of the network denotes the hierarchical number of significant correlations associated with each biomarker

After identifying the correlation profile of lymph-node and plasma compartments, highlighting the nuances of each compartment, we next investigated the associations between the systemic and local inflammatory profiles in EPTB. To do so, we employed a Spearman correlation matrix, using plasma and lymph-node biomarker levels (Fig. 3). Our analysis revealed lymph-node IL-6 as the top connected marker, showing correlations with plasma IL-7, TNF- β, IL-13, IL-6, IL-8, and IL-15. The second most highly connected marker was lymph node IL-1α, which showed positive correlations with plasma IL-7, TNF-β, IL-6, and IL-13. Of note, plasma TNF-β and IL-7 concentrations also demonstrated a higher number of correlations (Fig. 3).

Our results argue that there is a potential influence of plasma levels of TNF-β and IL-7 in the pathogenesis of lymph-node TB, defined by increased number of correlations between concentrations of these markers and critical pro inflammatory mediators in lymph-node, as IL-6 and IL-1 α.

### Correlation between CD4 counts, HbA1c values, and concentrations of inflammatory markers in plasma or lymph nodes

**Fig. 4:**
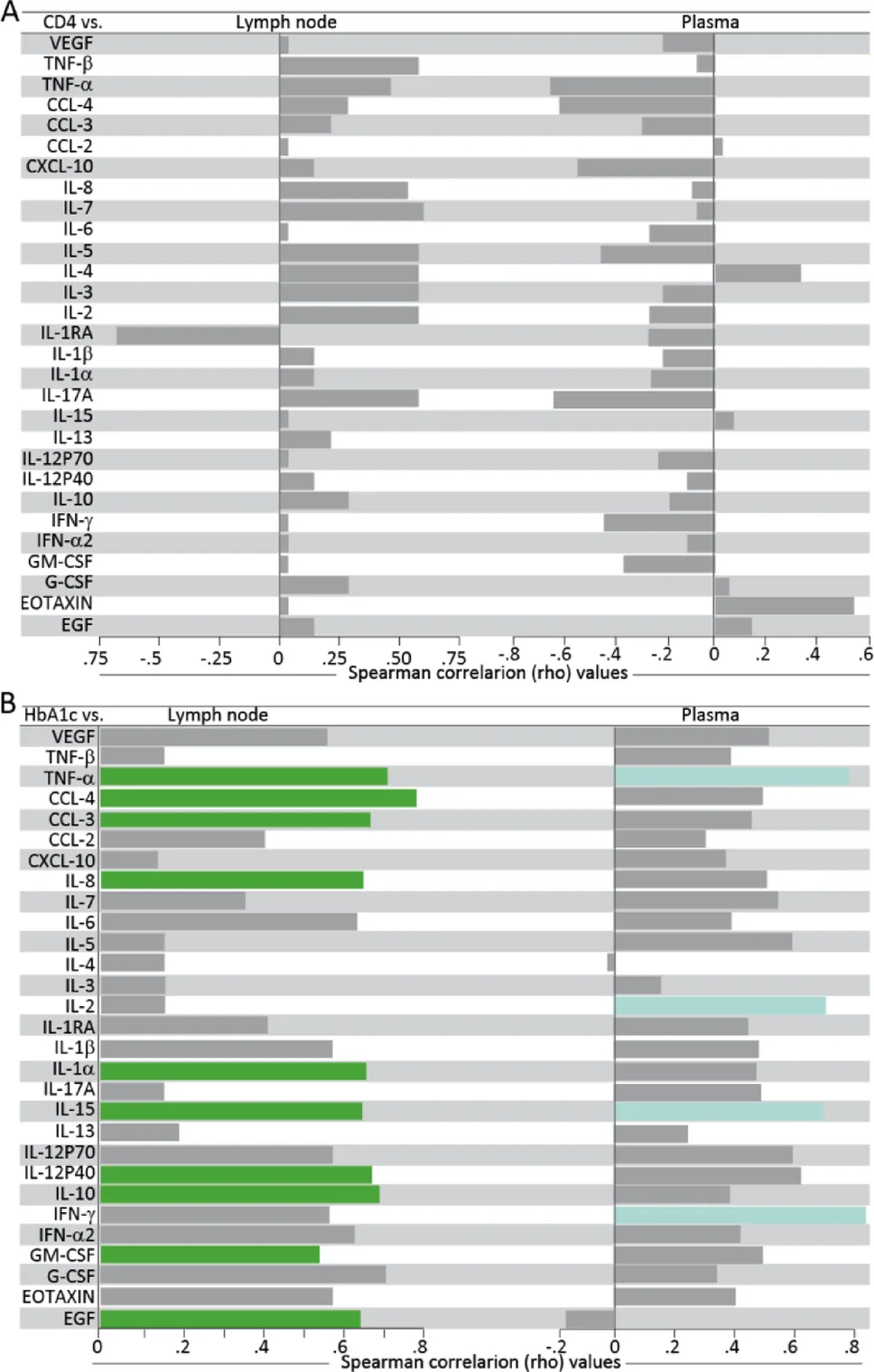
Influence of external factors on inflammatory activation in plasma and lymph node compartments. Spearman correlation analysis was used to test the associations between **A** CD4 count or **B** HbA1c percentage and plasma and lymph node levels of soluble inflammatory factors. Bars represent Spearman rank correlation coefficient (rho) values. Colored bars indicate statistically significant correlations (*P* < 0.05)

After characterizing immune activation in *Mtb* infection in both systemic and tissue compartments, we further evaluated the influence of host factors known to modulate TB immunopathology, namely HIV infection and glycemic control. Spearman correlation analyses were performed using CD4⁺ T-cell counts and HbA1c levels in relation to concentrations of soluble inflammatory factors measured in plasma and lymph node samples (Fig. 4).

No significant correlations were observed between CD4⁺ T-cell counts and inflammatory biomarker concentrations in either plasma or lymph node compartments (Fig. 4A), indicating that the inflammatory patterns identified in this cohort were largely independent of the degree of peripheral CD4⁺ T-cell depletion.

In contrast, HbA1c levels displayed multiple positive correlations with inflammatory mediators in both compartments, with a notably higher number and magnitude of associations at the tissue level (Fig. 4B). In lymph node samples, HbA1c positively correlated with TNF-α, CCL-4, CCL-3, IL-8, IL-1α, IL-15, IL-12p40, IL-10, GM-CSF, and EGF. In plasma, positive correlations were observed with TNF-α, IL-2, IL-15, and IFN-γ.

Collectively, these findings demonstrate that HbA1c levels were associated with inflammatory mediator concentrations in both compartments, particularly within lymph node tissue. Given the limited sample size and the fact that HbA1c measurements were available only for participants with tuberculous lymphadenitis, these findings should be interpreted as exploratory. Nevertheless, the greater number of HbA1c-associated correlations observed in lymph node samples is consistent with compartment-specific relationships between glycemic status and local inflammatory responses.

### A unique inflammatory profile characterizes lymph node tuberculosis

**Fig. 5:**
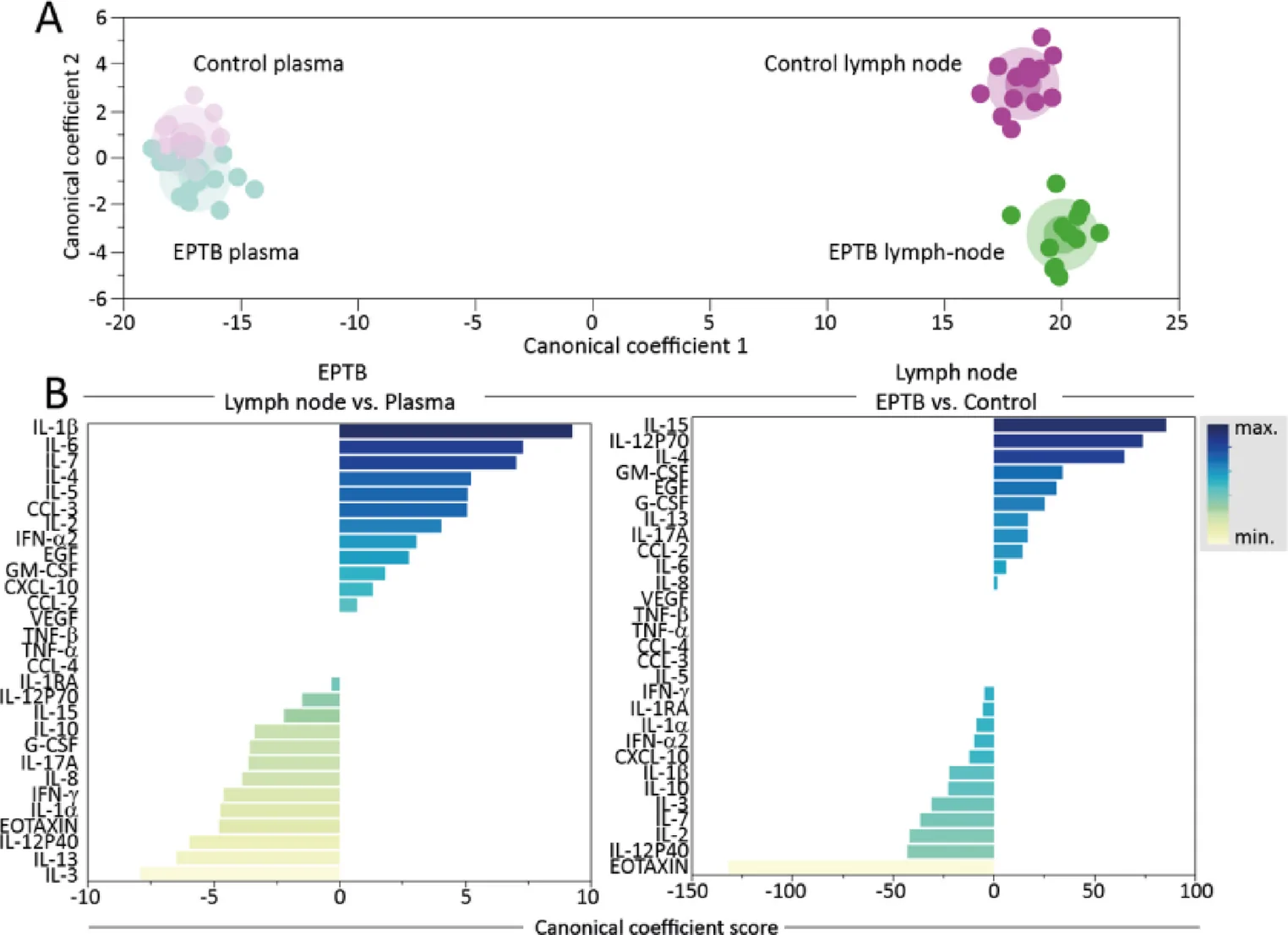
An Inflammatory Profile that Characterizes Lymph Node Tuberculosis. **A** In an exploratory approach, a sparse canonical correlation analysis (sCCA) was used to test whether experimental groups could be distinguished based on the overall expression profile of all the markers measured. **B** Canonical coefficient scores were calculated to identify the biomarkers responsible for the difference between groups in the sCCA model

We finally tested whether simultaneous measurement of inflammatory biomarkers in plasma and lymph node samples could distinguish the clinical groups. Given that we observed distinct correlation profiles among biomarker concentrations (Fig. 2), we applied a discriminant model using canonical correlation analysis, which considers the following: (1) number, (2) strength, and (3) identity of the biomarkers involved in correlations between the biomarker concentrations [16]. We found that the EPTB group, at the of lymph node level, could be completely distinguished from the other groups and compartments (Fig. 5A). Moreover, we plotted the canonical coefficient score of each biomarker included in the model, to identify the top markers responsible for the discrimination between the EPTB lymph node from EPTB plasma and control lymph node samples (Fig. 5B). In the comparison between the compartments, in EPTB group, we identified IL-1 β, IL-6, IL-7, IL-3, and IL-13 as the top markers in our model (Fig. 5B). When the concentrations of biomarkers at lymph node were compared between the clinical groups, the model identified IL-15, IL-12p70 and EOTAXIN contributed most for the discrimination (Fig. 5B). Together, these findings indicate that tuberculous lymphadenitis is associated with a distinct inflammatory architecture within lymph nodes, characterized by unique patterns of biomarker interactions and expression that differentiate affected tissues from both systemic circulation and non-TB controls.

## Discussion

In this study, we investigated inflammatory activation in paired plasma and lymph node samples from individuals undergoing diagnostic biopsy for pathological lymphadenopathy. We aimed to understand how immune responses are organized in tuberculous lymphadenitis. Our data demonstrated that EPTB is characterized by marked compartmentalization of inflammation, with limited systemic discrimination but profound tissue-level reorganization. Importantly, beyond differences in absolute cytokine concentrations, we observed substantial alterations in immune network architecture within infected lymph nodes, suggesting that lymph node TB is defined not only by inflammatory intensity but also by disruption of coordinated immune signaling at the site of disease.

Peripheral plasma biomarkers failed to distinguish EPTB from non-TB controls. In fact, hierarchical clustering and univariate analyses did not reveal statistically significant differences in systemic inflammatory mediators between groups. These findings reinforce the growing understanding that blood-based measurements, although practical and informative in pulmonary TB [8, 20], may not adequately capture immunological events occurring within infected tissues [5, 10]. In tuberculous lymphadenitis, the lymph node is not merely an immune surveillance organ but becomes an active infectious niche, structurally remodeled by granulomatous inflammation and necrosis [3, 7, 21]. It is therefore not surprising that systemic inflammatory signatures fail to fully reflect the immunological complexity unfolding at the site of disease.

In contrast, lymph node interstitial fluid revealed a tendency toward discrimination between EPTB and controls, characterized by increased IL-8, IL-3, and IL-5, and reduced IL-10 expression. Importantly, a sensitivity analysis restricted to participants with lymphoproliferative disorders yielded findings consistent with the primary analysis, supporting the conclusion that the observed cytokine profile was not driven by the inclusion of alternative infectious diagnoses within the control group. While these findings indicate differences in the inflammatory milieu at the tissue level, they do not allow definitive conclusions regarding the functional status of regulatory pathways. Instead, they suggest that immune responses in EPTB are organized differently within the lymph node microenvironment compared with systemic circulation. Although granulomas have traditionally been described as structured containment units [7, 21], accumulating evidence indicates that they are dynamic and heterogeneous structures. In this context, the relative reduction in IL-10 observed at the tissue level may reflect a shift in local immune balance. Whether this contributes to ineffective inflammatory control or altered tissue remodeling cannot be determined from our data, but it underscores that immune regulation within infected lymph nodes is likely more complex than what is captured by peripheral measurements alone.

Next, our univariate and Spearman correlation analyses provided additional insight into immune organization. Plasma samples from EPTB participants exhibited a dense and highly interconnected network, with IL-6, IFN-α2, and IL-12p70 acting as central nodes, suggesting coordinated systemic activation. In contrast, the lymph node network was markedly sparser. TNF-α and IL-1 members emerged as central nodes. Of interest, interactions between chemokines critical for cellular trafficking (CCL-2, CCL-3, CCL-4) and the regulatory cytokine IL-10 were limited. This pattern suggests a degree of uncoupling within the tissue inflammatory network.

Such disorganization may have important implications. Effective containment of Mtb depends not only on robust Th1 responses but also on coordinated signaling that integrates effector and regulatory pathways, avoiding tissue damage [4, 6, 7]. A sparse and poorly integrated network at the infection site may reflect altered immune communication within the granulomatous microenvironment. Rather than representing controlled containment, the infected lymph node may exhibit localized inflammatory amplification with limited regulatory coordination. These findings are consistent with altered immune organization at the site of disease, although the functional consequences of this network architecture cannot be inferred from the present data. Our cross-compartment analysis further revealed that systemic mediators such as TNF-β and IL-7 were strongly associated with pro-inflammatory signals within lymph nodes, including IL-6 and IL-1α. These findings suggest an association between systemic immune activation and local inflammatory dynamics. IL-7 plays a central role in T-cell survival and homeostasis [22], while TNF-β contributes to lymphoid tissue organization [23]. Their association with tissue-level pro-inflammatory mediators raises the possibility of biological interactions between systemic immune regulation and local immunopathology in EPTB, although the directionality of these associations cannot be determined from our cross-sectional data.

An additional and clinically relevant finding was the influence of glycemic control. A recent study showed that IL-18, GM-CSF, and CCL11 were linked to HbA1c levels [24]. However, those results reflected the interaction between glycemic control and systemic inflammatory responses, since was used cytokine plasma levels [24]. Here, our result reveals HbA1c levels correlated extensively with inflammatory mediators, particularly within lymph nodes. Positive associations with TNF-α, IL-1α, IL-8, IL-12p40, IL-15, GM-CSF, and other mediators indicate that glycemic status was associated with the local inflammatory profile observed in lymph node samples. Notably, these correlations were more pronounced locally than systemically, supporting the concept that metabolic factors may be linked to compartment-specific inflammatory responses in TB. However, these findings should be interpreted cautiously, as HbA1c measurements were only available for participants with tuberculous lymphadenitis and the sample size was limited. In contrast, CD4⁺ T-cell counts were not significantly correlated with inflammatory mediators, indicating that, in this cohort, tissue-level immune organization was not directly driven by peripheral CD4 count. This observation does not exclude a potential effect of immunosuppression, particularly HIV-associated immunosuppression, in TB pathogenesis. However, it may indicate that once lymph node infection is established, local inflammatory architecture becomes relatively independent of systemic CD4 counts.

The discriminant model further underscored the lymph node inflammatory signature in EPTB. Canonical correlation analysis clearly separated EPTB lymph nodes from both plasma samples and control lymph nodes. Key contributors to discrimination included IL-1β, IL-6, IL-7, IL-3, and IL-13 in the compartment-based comparison, and IL-15, IL-12p70, and EOTAXIN when distinguishing clinical groups. Importantly, discrimination was achieved by integrating the number and strength of biomarker interactions rather than relying solely on individual cytokine levels. This system-level approach aligns with the evidence that TB pathogenesis is defined by inflammatory architecture and immune trajectory rather than isolated mediators [11].

Our study has limitations, including the relatively small sample size and the heterogeneity within the control group, with lymphoproliferative disorders representing the largest diagnostic subgroup. Because these alternative causes of lymphadenopathy may themselves influence local inflammatory responses, the observed biomarker differences should be interpreted in the context of comparisons with clinically relevant differential diagnoses rather than normal, non-inflamed lymph node tissue. In addition, analyses involving HbA1c were restricted to participants with tuberculous lymphadenitis and should therefore be considered exploratory. Additionally, while interstitial fluid sampling provides valuable insight into tissue-level inflammation, it does not fully resolve cellular sources or spatial heterogeneity within granulomas. Nevertheless, the paired design and integrated network analysis strengthen the robustness of our study.

## Conclusion

Tuberculous lymphadenitis is characterized by pronounced compartmentalization of immune responses, with systemic inflammation remaining coordinated while tissue-level networks exhibit structural disorganization. Metabolic factors appear to preferentially modulate local inflammatory activation, and canonical network analysis reveals a distinctive inflammatory signature at the site of disease. These findings emphasize that EPTB cannot be fully understood through peripheral blood analyses alone and highlight the importance of tissue-specific immune profiling for advancing biomarker discovery and host-directed therapeutic strategies.

## Supplementary Information

Below is the link to the electronic supplementary material.

Supplementary Material 1

## Data Availability

Data supporting the findings of this study are available from the corresponding author upon reasonable request. The datasets generated and analyzed in this study, including spreadsheets containing Luminex measurements of soluble factors as well as clinical and demographic information of the study participants, are subject to approval by the local ethics committee. Access to these data will be granted in accordance with institutional and ethical regulations.
