# Supplementary material for: Compartment-specific inflammation in tuberculous lymphadenitis: a case-control study
Source: Inflamm Res. 2026 Sep 5;75(1):197. doi: 10.1007/s00011-026-02351-1 (PMC13546146; doi:10.1007/s00011-026-02351-1)
Supplement: Supplementary file 1 [file 11_2026_2351_MOESM1_ESM.docx]

**Supplementary Figure 1. Sensitivity analysis restricted to lymphoproliferative controls.** Concentrations of IL-10, IL-8, IL-3, and IL-5 were measured in lymph node interstitial fluid samples from participants with tuberculous lymphadenitis (EPTB) and from non-TB controls restricted to individuals with lymphoproliferative disorders, the largest diagnostic subgroup among controls. Each dot represents an individual participant. Horizontal bars and whiskers indicate the median and interquartile range. Comparisons between groups were performed using the Mann–Whitney U test, with adjusted p values shown in each panel. The direction of the associations observed in the primary analysis was maintained, with lower IL-10 and higher IL-8, IL-3, and IL-5 concentrations in EPTB samples compared with lymphoproliferative controls.
